# Gluten Vehicle and Placebo for Non-Celiac Gluten Sensitivity Assessment

**DOI:** 10.3390/medicina55050117

**Published:** 2019-04-26

**Authors:** Oscar Gerardo Figueroa-Salcido, Noé Ontiveros, Francisco Cabrera-Chavez

**Affiliations:** 1 Nutrition Sciences Academic Unit, Autonomous University of Sinaloa, Cedros y Calle Sauces S/N, Fraccionamiento Los Fresnos, Culiacán 80019, Sinaloa, Mexico; gerardofs95@hotmail.com; 2 Division of Sciences and Engineering, Department of Chemical, Biological, and Agricultural Sciences (DC-QB), University of Sonora, Navojoa 85880, Sonora, Mexico

**Keywords:** celiac disease, wheat, gluten, non-celiac gluten-sensitivity, diagnosis

## Abstract

Non-celiac gluten sensitivity (NCGS) is a syndrome characterized by gastrointestinal and extraintestinal manifestations triggered after gluten ingestion in the absence of celiac disease and wheat allergy. Because of the lack of biomarkers for NCGS diagnosis, the cornerstone for its assessment is a single- or double-blind placebo-controlled (DBPC) gluten challenge. However, there are some non-standardized points in the diagnostic approach proposed by the experts. This complicate comparisons among the results published by different research groups. The gluten vehicle and placebo must be indistinguishable from each other, which entails sensory and technological evaluations of the designed gluten vehicle and placebo products. At the moment, there is no standardized method for the preparation of the gluten vehicle and placebo for carrying out DBPC gluten challenges for NCGS assessment. This review focuses on the challenges that researchers have to face, either for the development of an accepted gluten vehicle and placebo or for identifying NCGS cases on the basis of DBPC gluten challenges.

## 1. Introduction

Wheat is one of the most consumed cereals in western countries [1], but some wheat components trigger the diseases encompassed under the term gluten-related disorders [2]. These diseases principally include celiac disease, wheat allergy, and the new clinical entity non-celiac gluten-sensitivity (NCGS) [3,4]. The diagnosis of the first two conditions can be supported by using blood-based diagnostic tests, whereas there is a lack of reliable biomarkers for the diagnosis of NCGS [4]. Thus, experts of gluten-related disorders have proposed that single- or double-blind placebo-controlled (DBPC) gluten challenges should be carried out to establish the diagnosis of NCGS, either for clinical practice (single-blind) or research purposes (double-blind) [5]. The challenges have to be carried out using cooked gluten, and the gluten vehicle and placebo must be indistinguishable from each other [5]. However, no study has proposed a gluten vehicle and placebo that meet the characteristics given by the experts. Consequently, there is a huge heterogeneity in the characteristics of the gluten vehicles and placebo used for carrying out DBPC gluten challenges for NCGS assessment [6,7]. This complicates comparisons among studies [8] and highlights the need of standardized gluten vehicle and placebo accepted by the scientific and clinical communities. In this narrative review, we present advances on the design of both gluten vehicle and placebo used for NCGS assessment.

## 2. DBPC Gluten Challenges

**Figure 1:**
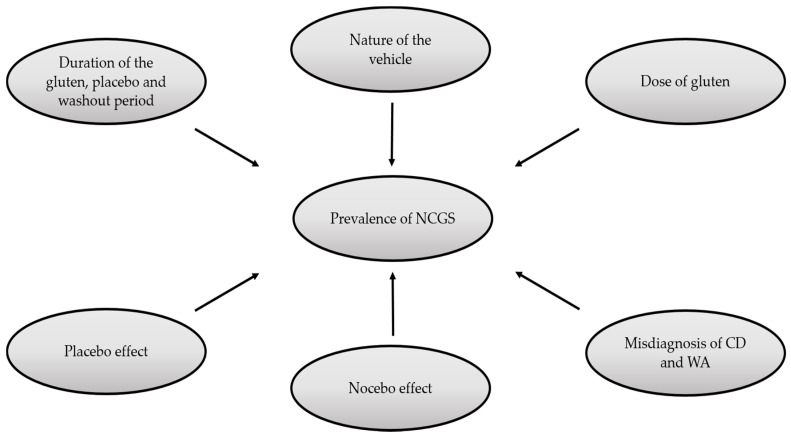
Factors that influence non-celiac gluten sensitivity (NCGS) assessment using double-blind placebo-controlled (DBPC) gluten challenges. CD: celiac disease, WA: wheat allergy.

There are some variables that should be taken into account when interpreting the outcomes of DBPC gluten challenges (Figure 1). The challenge, as stated in The Salerno experts’ criteria, involves two stages: (1) assessing the clinical response to a gluten-free diet (GFD) and (2) assessing the effect of reintroducing gluten after a period of treatment with the GFD [5]. In each stage, the patients use a self-administered instrument, which is called Gastrointestinal Symptom Rating Scale (GSRS). This instrument evaluates in a scale from 1 (mild) to 10 (severe) the gastrointestinal and extra-intestinal manifestations associated with NCGS [5]. In stage 1 (response to a gluten-free diet), a reduction >30% of the symptomatic baseline score for one to three main symptoms or at least one symptom with no worsening of the others is needed to pass to the second stage. In stage 2 (DBPC gluten challenge with crossover), data about the symptoms during the gluten and placebo challenge are collected. Differences in the scores of the GSRS of at least 30% are required to discriminate a positive response from a negative result [6]. However, some points in this diagnostic approach have not been carefully considered by some researchers. As shown in Table 1, the time periods reported for carrying out DBPC gluten-challenges vary from 1 day to 6 months, although challenges shorter than one week might not detect fluctuating symptoms [9].

Regarding the doses of gluten, these vary from 2 g/day to 52 g/day [10,11], and this variation could impact the symptomatic outcomes. In fact, Zanini et al. [12] estimated an NCGS prevalence rate of 34% using a vehicle containing 7.9 g of gluten and a 10-day gluten challenge, but others estimated half of that prevalence using a vehicle containing 4.7 to 5.6 g of gluten in a 7-day gluten challenge [7,8,13]. Besides the dose of gluten utilized for the challenges and the time of gluten exposure, another factor that deserves attention is the washout period. Some authors have recommended washout periods for more than one week when carrying out DBPC gluten challenges. This is to ensure specificity and to prevent fluctuating symptoms [8]. Overall, there is a huge heterogeneity regarding the parameters of time of gluten exposure, dose of gluten, and washout period utilized for carrying out the DBPC gluten challenge for NCGS assessment, either in clinical or research settings (Table 1).

Additionally, researchers and clinicians should take into account the placebo and nocebo effects, as the manifestations of symptoms during the gluten and placebo challenge may be similar [22]. In fact, the nocebo effect could be as high as 40% in DBPC studies [8]. On the side of the positive conditioning, the studies by Dale et al. [20] and by Skodje et al. [19] reported higher manifestations of symptoms during the placebo challenge (up to 37%) than during the gluten challenge (22%). In this context, it has been proposed that increasing the ratio placebo challenges/gluten challenges to 2:1 can be an effective strategy to minimize false-positive cases [23].

According to the Salerno experts, the characteristics of the gluten vehicle and placebo utilized for carrying out the DBPC gluten challenge should be as follows: “The gluten and placebo preparations must be undistinguishable in look, texture and taste, and balanced in fibers, carbohydrate, fat and possible protein” [5]. This implies a big challenge for food science technologists mainly due to the viscoelastic properties of gluten, which strongly impact on the texture and appearance of gluten-containing products. In fact, the technological and sensory properties provided by gluten are difficult to mimic in gluten-free products [24], and no study conducted to rule in or rule out NCGS has reported the use of a gluten vehicle and placebo that meet the characteristics given by the Salerno experts (Table 2). Thus, to identify NCGS cases, the design and the widespread use of a gluten vehicle and placebo that meet the characteristics proposed by the experts or of other gluten vehicle and placebo accepted by scientists and clinicians seem urgent.

## 3. Current State of NCGS

NCGS is defined as “a syndrome characterized by intestinal and extra-intestinal symptoms related to the ingestion of gluten-containing food, in subjects that are not affected by either celiac disease or wheat allergy” [5]. Although the real prevalence of NCGS remains unknown, current data suggest that it ranges from 0.6% to 6% in the general population [25,26,27,28,29]. One of the major challenges in the diagnosis of NCGS is the identification of the components that trigger the manifestations reported [9,30]. This is an area that remains poorly understood and has been the object of extensive research. Certainly, there are different components that may cause adverse reactions in NCGS patients, such as gluten, FODMAPs (fermentable oligo-, di-, monosaccharides, and polyols) and ATIs (amylase and trypsin inhibitors) [31]. According to some authors, NCGS individuals are sensitive to one or another, if not all, of these wheat components [32,33]. Furthermore, there is a huge spectrum of both gastrointestinal and extraintestinal manifestations [34]. In line with this, it is suggested that the pathogenesis of NCGS should be drawn taking into account the collective effect of the wheat components [35]. In this case, the gluten vehicle and placebo utilized for carrying out DBPC gluten challenges for identifying NCGS cases should include standardized amounts of the main suspected triggers of the condition.

## 4. Characteristics of the Gluten Vehicle and Placebo for Carrying out DBPC Gluten Challenges

According to experts, the gluten vehicle for carrying out DBPC gluten challenges should contain cooked and homogeneously distributed gluten (8 g per dose) and the pro-inflammatory factor ATIs (0.3 g/8 g of gluten) and be FODMAPs-free [5]. The use of gelatin capsules is discouraged, and this has motivated the search for the best-suited gluten vehicle. However, changes in the content of gluten, ATIs, and FODMAPs modify the food matrix structure altering the sensory characteristics of the food. From a commercial point of view, the more similar the gluten-free product is to its gluten-containing counterpart the better it is, but for the purpose of a gluten vehicle and placebo indistinguishable from each other for carrying out DBPC gluten challenges, this is not the rule. The development of a gluten vehicle could start from a gluten-free base formulation, trying to preserve the characteristics when gluten is added. To exclude variations of some of the sensory characteristics, drying and milling of the gluten vehicle and placebo until a flour-like material is obtained could be helpful [36]. Certainly, the challenge for developing an appropriate vehicle for gluten administration and its respective placebo is to maintain them indistinguishable from each other, while keeping the placebo in gluten-, ATIs-, and FODMAPs-free conditions.

### 4.1. Sensory and Technological Characteristics Given by Gluten to Baked Food Products

Gluten represents 80–85% of the total protein from wheat [37] and includes two main subgroups of proteins: gliadin (alcohol-soluble fraction) and glutenins (weak acid-soluble fraction) [38]. These two proteins are responsible for many technological properties of baked food products. For instance, hydrated gliadins contribute mainly to the viscosity and extensibility of the dough, and hydrated glutenins confer cohesive and elastic properties, which are responsible for dough strength and elasticity [39]. Furthermore, interactions between both gliadins and glutenins increase dough viscosity and, at the same time, decrease the high level of elasticity conferred by glutenins. This balance between gliadins and glutenins are determinant for dough rheology [40] and, consequently, some additives are used in the preparation of most gluten-free baked goods to mimic the technological characteristics given by gluten. Thus, as shown in Table 1, some authors evaluate the sensory and/or technological characteristics of a designed gluten-free product and compare it with the same product without gluten additives instead of comparing it with the gluten-containing product, which is the one being surrogated. Others have reported sensory evaluations of both gluten vehicle and placebo to ensure that they were indistinguishable from each other (Table 1). However, the specific formulations and methods of preparation have not been reported, making it difficult to replicate the results obtained by different research groups.

### 4.2. Sensory and Technological Characteristics Given by FODMAPs to Baked Food Products

FODMAPs are a group of components encompassing oligosaccharides (fructo-oligosaccharides), disaccharides (lactose), monosaccharides (fructose), and polyols (sorbitol, mannitol, maltitol, xylitol, polydextrose, and isomalt). The consumption of FODMAPs may trigger adverse reactions in susceptible individuals [41]. The mechanisms underlying the symptoms triggered by FODMAPs can be categorized as follows: (1) poor absorption of fructose, polyols, and lactose in the small intestine, (2) activation of an osmotic effect due to the small size of FODMAPs and stimulation of mechanoreceptors that increase luminal water content, and (3) a high rate of FODMAPs fermentation by bacteria [42,43]. Certainly, FODMAPs are part of the suspected dietary components that can trigger the gastrointestinal symptoms seen in NCGS cases [10]. Therefore, the Salerno experts’ criteria established that the vehicles used for carrying out DBPC gluten challenges have to be FODMAPs-free, but just a few studies have reported the use of a FODMAPs-free placebo (Table 1). Understanding the role of FODMAPs in the food matrix of baked food products is important for designing a gluten vehicle and placebo with potential to be indistinguishable from each other.

The disaccharide sucrose (glucose plus fructose) can interact with other food ingredients and modify the sensory and physical properties of food products, such as sweetness, flavor, color formation, and texture [44]. Because sucrose provides more sweetness than other mono- and di-saccharides, such as maltose and glucose, its presence can increase the sweet flavor in many cereal-based products [45]. Furthermore, sucrose is important for the aeration of baked products, a process that increases the volumetric aeration rate and decreases the bubble size [46]. On the other hand, oligosaccharides (saccharides containing 3–10 sugar moieties) confer low-intensity sweetness to foods [47] but provide increased viscosity, improving the body and mouthfeel of food products [48]. In addition, oligosaccharides and other sugars are important for browning intensity throughout Maillard reactions and provide a high moisture-retaining capacity, preventing excessive drying [48]. For the purposes of DBPC gluten challenges for NCGS assessment, both gluten vehicle and placebo have to be FODMAPs-free. Thus, the technological and sensory characteristics given by FODMAPs to baked foods would not be of relevance in the design of a gluten vehicle and placebo, but researchers should take into account that the absence of both FODMAPs and gluten in a food matrix makes more feasible the design of a gluten vehicle and a placebo different from a bakery product.

### 4.3. Sensory and Technological Characteristics Given by ATIs to Baked Food Products

ATIs are a group of low-molecular-weight proteins (~15 kDa) that have amylase and trypsin inhibitory properties and represent ~4% of the total protein content in wheat flour [33,49]. They have been proposed as the potential triggers of the manifestations seen in NCGS cases [50]. ATIs from wheat, rye, and barley, but not from other plant species, can activate the innate immune system throughout their interaction with toll-like receptor 4, giving rise to TLR4-MD2-CD14 complexes and promoting the release of proinflammatory cytokines by myeloid cells [51,52]. Amylases are used mainly in fermented bakery products. These enzymes degrade the flour starch into dextrins allowing dough fermentation by yeasts. This process improves the volume of fermented breads and crumb texture [53]. Furthermore, amylases catalysis increases the content of fermentable and reducing sugars in flour, promoting the formation of Maillard reactions, which intensify bread flavor and crust color [54]. Regarding trypsin, it reduces the mixing time of flour dough and improves some sensory properties of bakery products, such as texture, flavor, and crust color of a bread loaf [55,56]. Thus, the sensory and technological characteristics given by ATIs to baked foods other than fermented bread seems not to be as relevant as those given by gluten and FODMAPs. However, it remains to be evaluated in detail whether the presence or absence of 0.3 g of ATIs/8 g of gluten involves significant changes of the technological and sensory characteristics of baked food products.

## 5. Perspectives

Although anti-gliadin IgG [57,58] and anti-nucleus antibodies seem to be associated with NCGS [58,59], there is still a lack of highly sensitive and specific serological biomarkers for NCGS, and the most accepted diagnostic algorithm for this clinical entity includes DBPC gluten challenges as the cornerstone. There are some non-standardized points in such an approach, which complicate fair comparisons among studies focused on the identification of NCGS. The most notable of these points is the lack of a gluten vehicle and a placebo that meet the characteristics given by the Salerno experts. The gluten vehicle and placebo must be indistinguishable from each other. In this context, sensory evaluations can be considered mandatory if the research purpose is the design of a gluten vehicle and a placebo for carrying out DBPC gluten challenges for NCGS assessment. Furthermore, the full recipe and method of preparation of the gluten vehicle and placebo should be clearly stated in order to promote the replication of the results and the acceptance of the proposed methodology by the scientific and clinical communities.

Biomarkers at intestinal level have been proposed to aid in the diagnosis of NCGS. On one hand, histological evaluations suggest that eosinophils (increased number) could be an intestinal biomarker for NCGS [60,61], while others have proposed the evaluation of mast cells, T helper, and intraepithelial lymphocytes [62]. On the other hand, expression analyses of immune molecules such as tissue transglutaminase 2, interferon gamma, toll-like receptor 2, and myeloid differentiation factor 88 are less promising as a diagnostic tool [63], although these analyses are of relevance to elucidate the pathogenic mechanisms that underlie NCGS [64]. The main limitation of these tests is that they include an invasive procedure. The tests are reliable in patients on a gluten-containing diet, but standardized gluten challenges should be carried out for patients already following a gluten-free diet. For instance, a three-day gluten challenge with cooked and homogeneously distributed gluten can trigger T cell immunity to gluten without causing significant architectural disruption to the small intestinal mucosa in celiac disease cases, but they do not trigger T cell immunity to gluten in NCGS individuals [65,66]. Because of the invasive nature of intestinal biomarkers evaluation, biopsy-proven NCGS assessment should only be considered in well-established symptomatic cases of gluten intake. These cases should be identified on the basis of DBPC gluten challenges, carried out using a gluten vehicle and a placebo indistinguishable from each other. As mentioned before, such gluten vehicle and placebo are still to be developed, but we believe that the conditions exist for a close collaboration between cereal technologists and clinical researchers for developing accepted gluten vehicle and placebo for NCGS diagnosis purposes.

## Figures and Tables

**Table 1:** Studies reporting DBPC gluten challenge results for NCGS assessment. GSRS-IBS: Gastrointestinal Symptom Rating Scale-Irritable bowel syndrome

Study/Design	No. Patients	Presentation/Dose of Gluten	Placebo Vehicle	Gluten Challenge	Washout Period	Outcomes
Biesiekierski et al., 2011—DBPCT [14]	34	Bread and muffin, 16 g/day	Bread and muffin	6 weeks	-	13 patients (68%) in the gluten group reported symptoms that were not adequately controlled, but 6 patients (40%) in the placebo group reported the symptoms.
Biesiekierski et al., 2013—DBPCT with cross-over [10]	37	Diet High: 16 g/day Low: 2 g/day	16 g whey protein	1 week	Up to 2 weeks	The symptoms improved in all the participants (n = 37) during a low-FODMAP diet, although adverse reactions to gluten were reported by 8% (n = 3) of the patients.
Peters et al., 2014—DBPCT with cross-over [15]	22	Diet 16 g/day	16 g whey protein or placebo	3 days	At least 3 days	Gluten ingestion was associated with a high overall symptoms-based score of depression (63%; n = 14) in comparison to the ingestion of placebo (36%; n = 8).
Shahbazkhani et al., 2015—DBPCT [16]	72	Gluten meal powder, 52 g/day	Gluten-free meal powder (rice flour, corn starch, and glucose)	6 weeks	-	The symptoms were better controlled in the placebo group than in the group that received gluten (83.8%; n = 31 and 25.7%; n = 9, respectively).
Di Sabatino et al., 2015—DBPCT with cross-over [13]	61	Gastrosoluble capsules, 4.375 g/day	Gastrosoluble capsules, 4.375 g/day rice starch	1 week	1 week	Gluten intake significantly increased the overall symptoms in comparison to the placebo group. However, only three patients were defined as NCGS subjects (5%).
Zanini et al., 2015—DBPCT with cross-over [12]	35	Flour, 7.9 g/day	Flour, 7.67 starch, 0.68 g lactose, 0.01 g fructans	10 days	2 weeks	12 (34%) out of 35 participants were classified as NCGS patients, but 17 (49%) of the participants reported symptoms during the placebo challenge.
Elli et al., 2016 DBPCT with cross-over [7]	98	Gastrosoluble capsules, 5.6 g/day	Gastrosoluble capsules, 5.6 g/day rice starch	1 week	1 week	28 out of 98 patients reported symptomatic relapse during gluten ingestion. However, only 14% of those that reacted to gluten ingestion did not respond to the placebo challenge.
Picarelli et al., 2016-DBPCT with cross-over [17]	26	Crosssaint, 10 g/day	Gluten-free croissant	1 day	-	Eight (61.5%) out of 13 NCGS patients reported a high overall score of symptoms during the gluten challenge. However, in a second group, 6 (46.2%) out of 13 NCGS patients reported symptomatic relapse during the placebo challenge.
Rosinach et al., 2016 [18]-DBPCT	18	Sachets, 16.2 g/day	Gluten-free sachets	6 months	-	Ten (91%) out of 11 participants reported adverse reactions after gluten ingestion. In a second group, only two (28.5%) out of seven participants reported symptomatic relapse after placebo ingestion.
Skodje et al., 2018 [19]—DBPCT with cross-over	59	Muesli bar, 5.7 g/day	2.1 g fructan/placebo muesli bar	1 week	At least 7 days	Symptomatic response in the placebo group (37.28%) was higher than in the group that received gluten (22.03%). The overall GSRS-IBS score was higher in the fructan group (44%) than in the group that received gluten (22%). However, no significant difference in the overall GSRS-IBS score between the fructan group (44%) and the placebo group (37%) was found.
Dale et al., 2018 [20]—DBPCT with cross-over	20	Muffin, 11 g/day	Gluten-free muffin	4 days	3 days	Only 4 out of 20 patients were diagnosed with NCGS (20%). Patients that did not meet the criteria for NCGS (80%) reported more severe symptoms with placebo than with gluten.
Francavilla et al., 2018 [21]—DBPCT with cross-over	28	Sachets, 10 g/day	Gluten-free starch sachets	2 weeks	1 week	Eleven (39.3%) out of 28 patients were classified as NCGS cases.

**Table 2:** Characteristics of the gluten vehicle and placebo proposed by the Salerno experts and the characteristics proposed by others.

Study	Presentation	Gluten		ATIs		FODMAP		Protein		Fat		HC	
		GV	PV	GV	PV	GV	PV	GV	PV	GV	PV	GV	PV
Salerno experts Catassi et al., 2015 [5]	It can be a muesli bar, bread, muffin or may differ between children and adults.	8 g/day	Free	0.3 g/8 g gluten	Free	Free	Free	Possible balanced		Balanced		Balanced	
Elli et al., 2016 [7]	Gastrosoluble capsules	5.6 g/day	Free	NR	NR	NR	Low-FODMAP	NR		NR		NR	
Rosinach et al., 2016 [18]	Sachets	16.2 g/day	Free	NR	NR	Free	Free	Not balanced		Not balanced		Not balanced	
Skyje et al., 2018 [19]	Muesli bar	5.7 g/day	Free	Free	Free	Free	Free	Possible balanced		Balanced		Balanced	
Dale et al., 2018 [20]	Muffin	11 g/day	Free	NR	NR	Low-FODMAP	Low-FODMAP	Possible balanced		Balanced		Balanced	
Francavilla et al., 2018 [21]	Sachets	10 g/day	Free	0.4g	Free	Free	Free	Possible balanced		Balanced		Balanced	

Acronyms used: GV: gluten vehicle, PV: placebo vehicle, ATIs: amylase and trypsin inhibitors, FODMAP: fermentable oligo-, di-, monosaccharides, and polyols, HC: Carbohydrates, NR: not reported.
